# Perspectives of Canadian Plastic Surgeons on the Surgical Management of Pressure Injuries

**DOI:** 10.1177/22925503241308541

**Published:** 2024-12-22

**Authors:** Erika Opingari, Romain Laurent, Ho Yee Wan, David L. Wallace, Alan D. Rogers

**Affiliations:** 1 Ross Tilley Burn Centre, Sunnybrook Health Sciences Centre, Toronto, ON, Canada; 2 Division of Plastic and Reconstructive Surgery, Department of Surgery, University of Toronto, Toronto, ON, Canada

**Keywords:** pressure injury, plastic surgery, surgical management, flap coverage, multidisciplinary care, lésion de pression, chirurgie plastique, prise en charge chirurgicale, couverture du lambeau, soins multidisciplinaires

## Abstract

**Introduction:** Pressure injuries, particularly among patients with spinal cord injuries and the elderly, significantly contribute to morbidity, mortality, and financial sequelae. Surgical interventions, including debridement and flaps, may improve outcomes, especially for stage 3 and 4 pressure injuries. This survey assesses Canadian plastic surgeons’ perspectives and practices regarding the surgical management of these injuries. **Methods:** A web-based survey was distributed to 405 registered members of the Canadian Society of Plastic Surgeons (CSPS) and remained open for a 6-week period. The 16-question survey explored management practices for pressure injuries and the training associated with resident education. **Results:** Responses from 129 surgeons (31.8%) were analyzed. Of these, 85% manage stage 3 or 4 pressure injuries, though only 67.5% performed both debridement and flap coverage. The majority believe care should ideally occur in community facilities with plastic surgery coverage rather than being centralized in academic centers, although careful patient selection, perioperative planning, and rehabilitation are critical to justify the surgical investment. Most respondents (83%) agreed that plastic surgery residents should be proficient in both debridement and flap coverage by the end of their training. **Conclusion:** The survey indicates that most surgeons prefer managing pressure injuries in facilities with adequate plastic surgery support rather than exclusively at academic centers. However, structured management pathways and enhanced training in pressure injury care remain crucial. Future healthcare policy and research should focus on improving care delivery and patient outcomes, ensuring that all facilities involved in this care are equipped with the necessary resources and multidisciplinary expertise.

## Résumé

**Introduction :** Les lésions de pression, notamment chez les patients ayant des lésions du rachis et chez les personnes âgées, contribuent de manière appréciable à la morbidité, à la mortalité et aux séquelles financières. Les interventions chirurgicales, y compris les débridements et les lambeaux, peuvent améliorer les résultats cliniques, surtout en présence de lésions de pression de stade trois et quatre. Le présent sondage évalue les points de vue et les pratiques des plasticiens canadiens à l’égard de la prise en charge chirurgicale de ces lésions. **Méthodologie :** Les chercheurs ont distribué un sondage en ligne à 405 membres inscrits de la Société canadienne des chirurgiens plasticiens (SCCP), lequel est demeuré accessible pendant six semaines. Le sondage de 16 questions portait sur les pratiques de prise en charge des lésions de pression et de la formation liée à l’enseignement des résidents. **Résultats : ** Les chercheurs ont analysé les réponses de 129 chirurgiens (31,8 %). De ce nombre, 85 % prenaient en charge des lésions de pression de stade trois ou quatre, mais seulement 67,5 % effectuaient à la fois des débridements et des couvertures par lambeau. La majorité était d’avis qu’idéalement, les soins devraient être prodigués dans des établissements communautaires qui fournissent des services de chirurgie plastique plutôt que d’être centralisés dans des centres universitaires, mais qu’une sélection attentive des patients, une planification périopératoire et une réadaptation sont essentielles pour justifier un investissement en chirurgie. La plupart des répondants (83 %) convenaient que les résidents en chirurgie plastique devraient être habiles en débridement et en couverture par lambeau à la fin de leur formation. **Conclusion :** D’après le sondage, la plupart des chirurgiens préfèrent prendre en charge les lésions de pression dans des établissements dotés d’un soutien approprié en chirurgie plastique plutôt qu’exclusivement dans les centres universitaires. Cependant, il demeure capital d’offrir des parcours de prise en charge structurés et des formations améliorées sur les lésions de pression. Les futures politiques et recherches en matière de santé devraient porter sur l’amélioration de la prestation des soins et les résultats cliniques des patients, afin que tous les établissements qui participent à ces soins soient pourvus des ressources nécessaires et des compétences multidisciplinaires.

## Introduction

Pressure injuries, previously known as pressure ulcers, pressure sores, decubitus ulcers, or bedsores, occur due to prolonged contact over bony prominences, potentially leading to significant tissue necrosis, osteomyelitis, sepsis, and even death.^1,2^ These injuries are particularly prevalent among patients with limited mobility, such as those with spinal cord injuries and elderly individuals. The National Pressure Injury Advisory Panel (NPIAP) classifies pressure injuries into 4 stages, with stages 3 and 4 representing the most severe, involving full-thickness skin loss and potential exposure of muscle or bone.^3^

Pressure injuries pose substantial healthcare challenges in Canada. Treatment costs for stage 4 pressure injuries can be as high as 90,000 Canadian Dollars per case, creating a significant financial burden.^4^ Beyond financial implications, these injuries are associated with increased morbidity, higher mortality rates, and psychosocial and economic consequences for patients and their families.^5^ For advanced-stage pressure injuries, surgical management—including debridement and flap reconstruction—may play a critical role in preventing complications and promoting wound healing in suitable candidates.

Despite this, limited data exists on the perspectives and practices of Canadian plastic surgeons regarding the management of pressure injuries. This survey seeks to understand the current practices in managing stage 3 and 4 pressure injuries, the infrastructure in place, and to explore whether surgical training adequately prepares residents for these procedures.

## Methods

A web-based survey was created and distributed via SurveyMonkey (Momentive, San Mateo, California) to 405 registered members of the Canadian Society of Plastic Surgeons (CSPS). The survey consisted of 16 questions, incorporating multiple-choice and open-ended formats, to assess participants’ experiences with managing pressure injuries, preferred surgical techniques, perceived barriers, and views on resident training (see Supplementary Appendix).

The survey remained open for 6 weeks, with reminder emails sent on 2 occasions to maximize participation. Results were analyzed using descriptive statistics, with categorical variables presented as frequencies and percentages, and continuous variables as means and standard deviations or medians with interquartile ranges.

## Results

A total of 129 plastic surgeons responded to the survey, yielding a response rate of 31.8%. Table 1 summarizes the responses. Most respondents practiced in Ontario (43.4%), followed by British Columbia (17%), Alberta (12.4%), and Quebec (9.4%). A large proportion of respondents (43.4%) had been in practice for over 20 years, and 80% had completed fellowship training, with microsurgery (40.3%) and hand surgery (31.8%) as the most common sub-specialties.

**Table 1.** The Multiple Choice Questions and the Most Frequently Selected Answers.

Question	Most popular response	Second most popular response	Third most popular response
Please select the province in which your primary practice is located.	Ontario (43.4%)—56 responses	British Columbia (17%)—22 responses	Alberta (12.4%)—16 responses
How many years have you been in practice since qualifying as a plastic surgeon?	>20 years (43.4%)—56 responses	5-10 years (17%)—22 responses	0-5 years (14%)—18 responses
Did you complete a formal training fellowship after qualifying as a plastic surgeon?	Yes—microsurgery (40.3%)—52 responses	Yes—hand surgery (31.8%)—41 responses	No (15.5%)—20 responses
Are pressure sores referred to your practice?	Yes—sometimes (54.3%)—70 responses	Yes—often (30.2%)—39 responses	No—Never (15.5%)—20 responses
If pressure sores are referred to your practice, do you usually:	Debride and perform the flap if the patient is stable (67.5%)—87 responses	Assess and debride if required before referral (10.9%)—14 responses	Pressure sores are not referred to me (10.9%)—14 responses
If you debride but don’t perform flap coverage, why don’t you perform flap surgery if indicated?	I usually perform the flap surgery (53.5%)—69 responses	I do not offer these patients surgery (30.23%)—39 responses	I do not debride pressure sores in my practice (8.53%)—11 responses
If you perform flap surgery for patients with pressure sores, which areas do you cover?	All of the above (65.89%)—85 responses	I do not do these surgeries (30.23%)—39 responses	Ischial only (3.88%)—5 responses
If you perform flap surgery for pressure sores, which flaps do you use?	Either myocutaneous or fasciocutaneous depending on patient (55%)—71 responses	I do not do these surgeries (29.5%)—38 responses	Myocutaneous usually (8.5%)—11 responses
If you perform flap surgery for pressure sores, which type of flap do you perform?	I do a variety of different flap surgeries depending on the patient (63.6%)—82 responses	I do not do these surgeries (29.5%)—38 responses	Usually rotation flap (5.4%)—7 responses
If you perform flap surgery for pressure sores, do you:	Often debride in 1 surgery and do the flap in the next (32.6%)—42 responses	I do not do these surgeries (27.1%)—35 responses	Do both in the same surgery (25.6%)—33 responses
In terms of operating time for pressure sores, do you usually:	Use elective time at your discretion (49.6%)—64 responses	I do not operate on these patients (27.9%)—36 responses	Use emergency operating time (12.4%)—16 responses
Do you believe that pressure sores should be managed at the following facilities	Any facility with plastic surgical coverage and the appropriate support (61.2%)—79 responses	Any hospital with a plastic surgeon (25.6%)—33 responses	Only specific academic hospitals should offer care (13.2%)—17 responses
How many patients with pressure injuries do you estimate that you manage with a flap per year?	0-5 surgeries (48.8%)—63 responses	0 surgeries (30.2%)—39 responses	5-10 surgeries (16.3%)—21 responses
With reference to Plastic Surgery Residents and their training:	Plastic surgery residents should be proficient in debriding and managing flaps (83%)—107 responses	Residents should at least be able to debride (14%)—18 responses	Only specialists should manage (3%)—4 responses

Approximately 30% of respondents reported frequently receiving referrals for pressure injuries, while 54.3% reported occasional referrals. Around 67.5% stated they performed both debridement and flap reconstruction when indicated. Among those performing flap coverage, the majority (66.4%) treated common sites, including the sacrum, ischium, and trochanteric regions.

Flap type preference varied, with 55% alternating between myocutaneous and fasciocutaneous flaps based on wound characteristics. Most surgeons (63.6%) utilized rotation or advancement techniques, tailoring the approach to the wound and patient circumstances. More respondents preferred performing debridement and flap closure as separate procedures (32.6%) rather than in a single surgery (25.6%).

Only 21% performed more than 5 pressure injury-related flap procedures annually, with 48.8% performing at least 1 but fewer than 5 per year, and 30.2% performing none. Most surgeons (49.6%) used elective OR time at their discretion, while 12.4% relied on emergency operating time.

A majority (61.2%) felt that any facility with adequate plastic surgery support should offer this service, provided the surgeon has the necessary experience and the facility has appropriate support systems. Only 13.2% believed that only academic hospitals should provide flap coverage for these patients.

Regarding resident training, 83% believed that plastic surgery residents should be proficient in both debridement and flap coverage for pressure injuries by the end of their training, though 14% felt the focus should be limited to debridement.

The open-text responses provided additional insights into the challenges, training requirements, and diverse practices in pressure injury management. The following 8 prominent themes emerged:

1. Resource and Infrastructure Limitations: Many respondents highlighted significant resource constraints in nontertiary settings, including limited rehabilitation facilities and postoperative care programs. The COVID-19 pandemic was noted to have further strained operating room access and postoperative support.
2. Appropriateness and Candidacy for Flap Surgery: Skepticism regarding the long-term efficacy of flap surgery was common. Respondents noted high recurrence rates and patient noncompliance, suggesting that flap surgery should be reserved for select, ideal candidates committed to postoperative care.
3. Multidisciplinary Care Requirements: Successful pressure injury management, according to respondents, relies on a multidisciplinary approach involving physical therapy, occupational therapy, nutritional supplementation, social work, and discharge planning. Surgeons stressed that effective surgical outcomes depend heavily on postoperative support systems.
4. Resident Training and Exposure: Many respondents expressed concern about the limited exposure residents have to flap surgeries for pressure injuries. Many advocated for flap surgery for this indication to remain a core competency within plastic surgery training, ensuring residents graduate with confidence in managing these complex cases holistically as part of the team.
5. Importance of Aftercare and Patient Selection: The success of flap surgeries was noted to depend on both careful patient selection and postoperative compliance. Respondents emphasized that aftercare and a multidisciplinary approach often influence outcomes more than the surgical procedure itself.
6. Generalist versus Specialist Role in Pressure Injury Care: Views varied on whether general plastic surgeons should manage pressure injuries or if cases should be referred to specialists. Some saw management as within the scope of general practice, while others recommended referral to specialized centers for complex cases, especially given the lack of the required systems in many community settings.
7. Preference for Centers of Excellence: Some respondents suggested concentrating resources and expertise in specialized centers to improve patient outcomes. Although access to such centers was seen as beneficial, geographical and logistical barriers were acknowledged.
8. Role of Negative Pressure Wound Therapy (VAC): Many respondents favored negative pressure wound therapy (VAC) as a primary treatment or adjunct to flap surgery, particularly for patients who are poor surgical candidates. VAC was considered effective and resource-efficient compared to surgery.

These themes underscore diverse perspectives on pressure injury management among Canadian plastic surgeons. They highlight key challenges, including limited resources, training variability, and the need for a multidisciplinary approach. These insights suggest that tailored management strategies considering institutional capabilities and patient-specific factors are essential for optimizing outcomes in this challenging area.

## Discussion

This survey provides insight into the surgical management of pressure injuries among Canadian plastic surgeons. While many surgeons perform both debridement and flap coverage, barriers such as limited access to postoperative care and operating room availability are prevalent. These findings emphasize the need for improved healthcare infrastructure and policies that support optimal care delivery for patients with advanced-stage pressure injuries.

The respondents emphasized the importance of a multidisciplinary approach, involving plastic surgeons, wound care specialists, nutritionists, and physiotherapists, which is critical to comprehensive postoperative care.^6,7^ The survey indicates that inadequate postoperative care infrastructure is a key barrier, underscoring the need for comprehensive wound care teams to prevent complications and recurrence.^6−8^

A strong consensus exists that residents should be proficient in both debridement and flap coverage by the completion of their training. Similar to other areas in plastic surgery, simulation-based training may help bridge experience gaps, allowing residents to practice complex techniques in a controlled setting, thus enhancing their confidence and competency in managing advanced pressure injuries.^9^

While most respondents believe that pressure injuries should be managed in facilities with adequate plastic surgery support, 13.3% advocated for centralizing care to centers of excellence. Such centers could standardize pressure injury management, optimize outcomes through standardized protocols, and provide a robust training environment for future surgeons. Concentrating resources and expertise at these centers may improve perioperative planning, enhance postoperative rehabilitation, and offer a higher chance of surgical success.^6^

Given the substantial financial burden associated with managing advanced pressure injuries, healthcare policy should prioritize prevention strategies and ensure equitable access to surgical care. An emphasis on developing resources and follow-up care may help reduce the lifetime costs associated with managing these complex cases.

This study has several limitations that must be considered. The response rate of 31.8% may limit the generalizability of results and introduce a potential for nonresponse bias, where the views of nonrespondents might differ from those of participants. Additionally, selection bias may be present, as surgeons more engaged in wound management or academic training may have been more likely to participate in the survey. This could skew results, potentially underrepresenting those who perform fewer pressure injury-related surgeries. Future studies should aim to increase participation and capture a broader range of practices to provide a more comprehensive understanding of pressure injury management across Canada, along with the human and infrastructural resources required to offer acceptable care for this often-neglected patient population.

## Conclusion

This study provides valuable insights into the current practices of Canadian plastic surgeons in managing pressure injuries, underscoring the need for standardized care pathways, improved resident training, and healthcare infrastructure reforms. Establishing centers of excellence may optimize outcomes for patients with advanced-stage pressure injuries.

## Supplemental Material

sj-docx-1-psg-10.1177_22925503241308541 - Supplemental material for Perspectives of Canadian Plastic Surgeons on the Surgical Management of Pressure Injuries Supplemental material, sj-docx-1-psg-10.1177_22925503241308541 for Perspectives of Canadian Plastic Surgeons on the Surgical Management of Pressure Injuries by Erika Opingari, MD, Romain Laurent, MD, Ho Yee Wan, MD, PhD, David L. Wallace, MD, MSc, and Alan D. Rogers, MBChB, MMed, MSc in Plastic Surgery
