# Supplementary material for: Perspectives of Canadian Plastic Surgeons on the Surgical Management of Pressure Injuries
Source: Plast Surg (Oakv). 2024 Dec 22;34(1):130–4. doi: 10.1177/22925503241308541 (PMC11664560; doi:10.1177/22925503241308541)
Supplement: sj-docx-1-psg-10.1177_22925503241308541 - Supplemental material for Perspectives of Canadian Plastic Surgeons on the Surgical Management of Pressure Injuries [file sj-docx-1-psg-10.1177_22925503241308541.docx]

Appendix: Survey on Plastic Surgeons and the Management of Pressure Injuries in Canada

Survey Questions

1. Please select the province in which your primary practice is located:
   - Alberta
   - British Columbia
   - Manitoba
   - New Brunswick
   - Newfoundland and Labrador
   - Nova Scotia
   - Ontario
   - Prince Edward Island
   - Quebec
   - Saskatchewan
   - Northwest Territories
   - Nunavut
   - Yukon
   - Other (please specify)
2. At which training program did you receive the bulk of your plastic surgery training?
3. How many years have you been in practice since qualifying as a plastic surgeon?
   - 0-5
   - 5-10
   - 10-15
   - 15-20
   - Over 20
4. Did you complete a formal training fellowship after qualifying as a plastic surgeon?
   - No
   - Yes - Hand surgery
   - Yes - Craniofacial surgery
   - Yes - Microsurgery
   - Yes - Burn surgery
   - Yes - Breast reconstruction
   - Yes - Pediatric plastic surgery
   - Yes - Cosmetic surgery
   - Other (please specify)

Additional Comments (optional)

1. Are pressure sores referred to your practice?
   - Yes - Sometimes
   - Yes - Often
   - No - Never

Additional Comments (optional)

1. If pressure sores are referred to your practice, do you usually:
   - Assess and refer to a colleague
   - Assess and debride if required, before referral to another colleague for coverage
   - Decline completely
   - Decline but suggest another colleague
   - Debride and perform the flap if the patient is a candidate for surgery
   - Pressure sores are not referred to me

Additional Comments (optional)

1. If you debride but don’t perform flap coverage, why don’t you perform flap surgery if indicated?
   - I usually refer to another plastic surgeon
   - I do not have the operating time available to perform the surgery
   - I do not have the training to perform the surgery
   - I do not have the interest to perform the surgery
   - I do not usually debride pressure sores in my practice
   - I usually do perform the flap surgery
   - I do not offer these patients surgery because the system is not in place to offer good postoperative care and rehabilitation

Additional Comments (optional)

1. If you perform flap surgery for patients with pressure sores, which areas do you cover?
   - Sacral only
   - Ischial only
   - Trochanteric only
   - Other areas (please specify)
   - All of the above
   - I do not do these surgeries

Additional Comments (optional)

1. If you perform flap surgery for pressure sores, which flaps do you use?
   - Myocutaneous usually
   - Fasciocutaneous usually
   - Either myocutaneous or fasciocutaneous, depending on the patient and the wound
   - I do not do these surgeries

Additional Comments (optional)

1. If you perform flap surgery for pressure sores, which type of flap do you perform?
   - Usually advancement
   - Usually rotation
   - A variety of different flap surgeries depending on the wound and the patient
   - I do not do these surgeries

Additional Comments (optional)

1. If you perform flap surgery for pressure sores, do you:
   - Always debride in one surgery and perform the flap in the next
   - Often debride in one surgery and perform the flap in the next
   - Usually do both at the same surgery
   - Debride but do not do the flaps
   - I do not do these surgeries

Additional Comments (optional)

1. In terms of operating time for pressure sores, do you usually:
   - Use elective time specially dedicated to this work
   - Use elective time at your discretion
   - Use emergency operating time
   - Operating time has to be specially arranged to operate on these patients
   - Other surgical specialists’ operating time is used to operate on these patients (e.g., orthopedics, spine, trauma, general surgery, etc.)
   - Patients with pressure sores should never receive surgery
   - I do not operate on these patients

Additional Comments (optional)

1. Do you believe that pressure sores should be managed at the following facilities:
   - Any facility with plastic surgical coverage should have a surgical pathway to manage these patients within their facility
   - Only specific academic hospitals should offer flap coverage for these patients. These should be regional centers of excellence, where all involved are trained and interested in the care of these patients, with standardized and evidence-based plans.
   - Any facility should offer this service, assuming the plastic surgeon has the experience and skill to perform the surgery and the support systems are in place. Otherwise, a referral pathway must be established.
2. How many patients with pressure injuries do you estimate that you manage with a flap per year?
   - 0
   - 0-5
   - 5-10
   - 10-20
   - More than 20

Additional Comments (optional)

1. With reference to Plastic Surgery Residents and their training:
   - Plastic Surgery Residents should be able to debride a pressure sore with confidence but not necessarily perform the flap. Residents who have not been involved in the care of patients with pressure sores should not qualify as plastic surgeons.
   - Plastic Surgery Residents should be able to debride and cover a pressure sore with a flap. Residents who have not been involved in the care of patients with pressure sores should not qualify as plastic surgeons.
   - Pressure sore management is not within the scope of general plastic surgery. Managing pressure sores surgically should be for fellowship training only (i.e., performed only by sub-specialists).
2. Please add any further comments you may have (e.g., specific referral pathways for these patients in your area, challenges you have experienced).
